# Brucea javanica oil emulsion plus supportive care for refractory advanced colorectal cancer: a pilot RCT protocol

**DOI:** 10.3389/fphar.2025.1610575

**Published:** 2025-07-21

**Authors:** Liyuan Fang, Yan Wang, Yuhang Fang, Runxi Wang, Yi Xie, Shuhan Yang, Suying Liu, Ying Zhang

**Affiliations:** ^1^Department of Oncology, Guang’anmen Hospital of the Chinese Academy of Traditional Chinese Medicine, Beijing, China; ^2^Graduate School, Beijing University of Chinese Medicine, Beijing, China

**Keywords:** colorectal cancer, Chinese herbal medicine, randomized controlled trial, BJOEI, progression-free survival

## Abstract

**Background:**

Colorectal cancer (CRC) is a significant contributor to global mortality. However, the existing therapeutic approaches often fall short of achieving favorable outcomes especially in metastatic CRC. Brucea javanica Oil Emulsion Injection (BJOEI) as adjuvant therapy also showed superiority for cancer treatment in clinical practice. This trial aims to gather preliminary data to inform a phase III clinical trial evaluating the efficacy and safety of BJOEI in combination with best supportive care (BSC) for patients with advanced colorectal cancer who are refractory to all existing therapies.

**Methods:**

The study is designed as a multicenter, randomized, and controlled clinical trial. 60 eligible participants will be randomly assigned to the experimental or control group in a ratio of 1:1. The experimental group will receive BJOEI and BSC, while the control group will undergo BSC. The treatment will cease upon disease progression or when toxicity becomes intolerable. Follow-up assessments will be scheduled every 2 months, continuing until the patient dies, is lost to follow-up, or reaches 12 months post-randomization. The main outcome measured will be progression-free survival (PFS). Additional outcomes to be evaluated are clinical symptoms, quality of life, and overall survival (OS). Detailed records of adverse events (AEs) will be maintained.

**Expected outcomes:**

To the best of our knowledge, this is the first study to investigate the use of Traditional Chinese Medicine as a monotherapy in patients with advanced colorectal cancer who have failed multiple lines of standard treatment.

**Trial registration:**

Clinicaltrials.gov, NCT05897749. Registered on 09 May 2023.

## Introduction

Malignant tumors represent the second leading cause of global mortality, posing a paramount public health concern. According to the latest 2020 global cancer burden data released by the International Agency for Research on Cancer, there were 19.29 million newly diagnosed cancer cases and 9.96 million deaths worldwide in 2020. CRC accounted for 10% of the global cancer incidence and a mortality rate of 9%, highlighting a formidable challenge in the global landscape of cancer prevention and control (Sung et al., 2021). CRC, characterized by its heterogeneous nature, often arises from the intricate interplay of genetic and environmental factors. Current research emphasizes the role of mutations in APC, CHEK2, MLH1, MUTYH, and PTEN in increasing the risk of CRC (Seagle et al., 2023). Over half of the new cases may be linked to modifiable risk factors such as smoking, excessive alcohol consumption, antibiotic usage, sedentary lifestyle, and obesity (Zhou et al., 2023; Wu J. et al., 2023; Iwasaka et al., 2023; Zhai et al., 2023). Approximately 25% of all patients with CRC receive a diagnosis of metastatic CRC (mCRC), and 25%–30% of patients with an initial diagnosis of stage I–III CRC eventually develop mCRC within 5 years (Mousa et al., 2015; Cartwright, 2012; Shah et al., 2016; Arnold and Stein, 2013). The clinical treatment of CRC is mainly surgical treatment, while chemotherapy is a widely used adjuvant treatment for patients with middle-advanced CRC. According to the literature, approximately 80% of all recurrences manifest within the initial 3 years after surgery, with 95% occurring within 5 years (Sargent et al., 2009; Seo et al., 2013). Systemic therapy for metastatic colorectal cancer (mCRC) typically begins with chemotherapy regimens that combine a fluoropyrimidine with either oxaliplatin or irinotecan. Although immunotherapy shows potential for treating advanced CRC, it faces challenges such as immunotherapy resistance and a limited population of beneficiaries (Adebayo et al., 2023; Tan and Sahin, 2021). For mCRC that progresses despite these treatments, three oral drugs have been researched and approved: regorafenib (Grothey et al., 2013), fruquintinib (Dasari et al., 2023), and trifluridine/tipiracil (TAS-102) (Mayer et al., 2015). These medications are considered standard later-line treatments and have been shown to extend survival in patients with resistant mCRC. However, the benefits are not universal, as evidenced by the FRESCO study, which reported an objective response rate of only 4.7% for fruquintinib monotherapy.

Traditional Chinese medicine exhibits a range of pharmacological effects and plays a therapeutic role in many critical aspects of diseases. Brucea javanica, a medicinal plant found throughout Asia in open areas and secondary forests (Ye et al., 2015), has demonstrated significant potential in this regard. Extracts from its fruit have been shown to possess anti-proliferative and pro-apoptotic properties on human carcinoma cells (Man et al., 2012).BJOEI comprises oil-soluble components formulated into a water-in-oil emulsion with refined Brucea javanica, purified lecithin, glycerol, and injectable water. Widely employed as an anti-tumor Chinese herbal injection, BJOEI has obtained approval from the China Food and Drug Administration (China drug approval number: Z 19993152) for the treatment of lung cancer, brain metastases from lung cancer, and digestive system tumors. Recent studies investigated cytotoxicity effects of Brucea javanica on tumor such as lung (Xie et al., 2021), liver (Chen et al., 2016), colorectal (Bagheri et al., 2018a), gastric (Li et al., 2020), esophageal (Qiu et al., 2019), and pancreatic cancers (Wu Y. et al., 2023). Primarily, it initiates the activation of caspase-8/9 and concurrently modulates apoptosis-associated proteins. This coordinated modulation leads to the initiation of apoptotic pathways in HT29 and HCT116 CRC cells. Additionally, it exerts a direct inhibitory effect on HCT116 CRC cell proliferation by facilitating cellular autophagy (Bagheri et al., 2018a; Bagheri et al., 2018b; Yan et al., 2015). Besides, BJOEI as adjuvant therapy also showed superiority in efficacy and safety for cancer treatment in clinical practice (Chen et al., 2022). BJOEI plus chemoradiotherapy may have positive effects on lung cancer, gastric cancer and CRC (Nie et al., 2012; Wang et al., 2021; Gao and Zhang, 2023). It is very important to improve quality of life and reduce incidences of some adverse effects compared with chemoradiotherapy alone. This study is principally aimed at acquiring preliminary insights that will underpin a phase III clinical trial focused on the assessment of BJOEI’s efficacy and safety, administered in conjunction with optimal supportive care, for patients afflicted with mCRC who exhibit resistance to all prevailing therapeutic options.

## Methods

### Study objectives

The primary objective of this study is to gather preliminary data to inform a phase III clinical trial evaluating the efficacy and safety of BJOEI in combination with best supportive care for patients with advanced colorectal cancer who are refractory to all existing therapies.

### Study design

**FIGURE 1:**
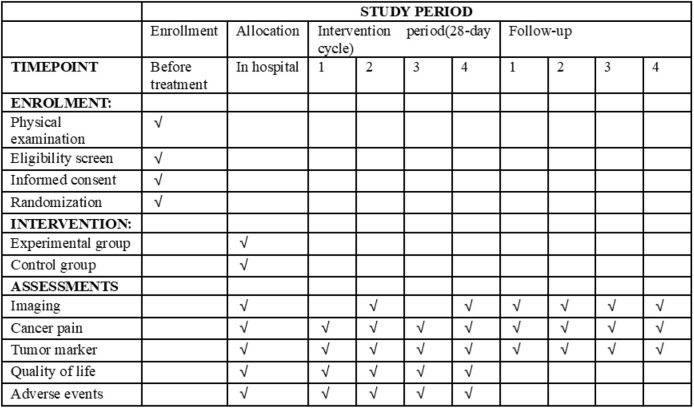
Participant timeline.

**FIGURE 2:**
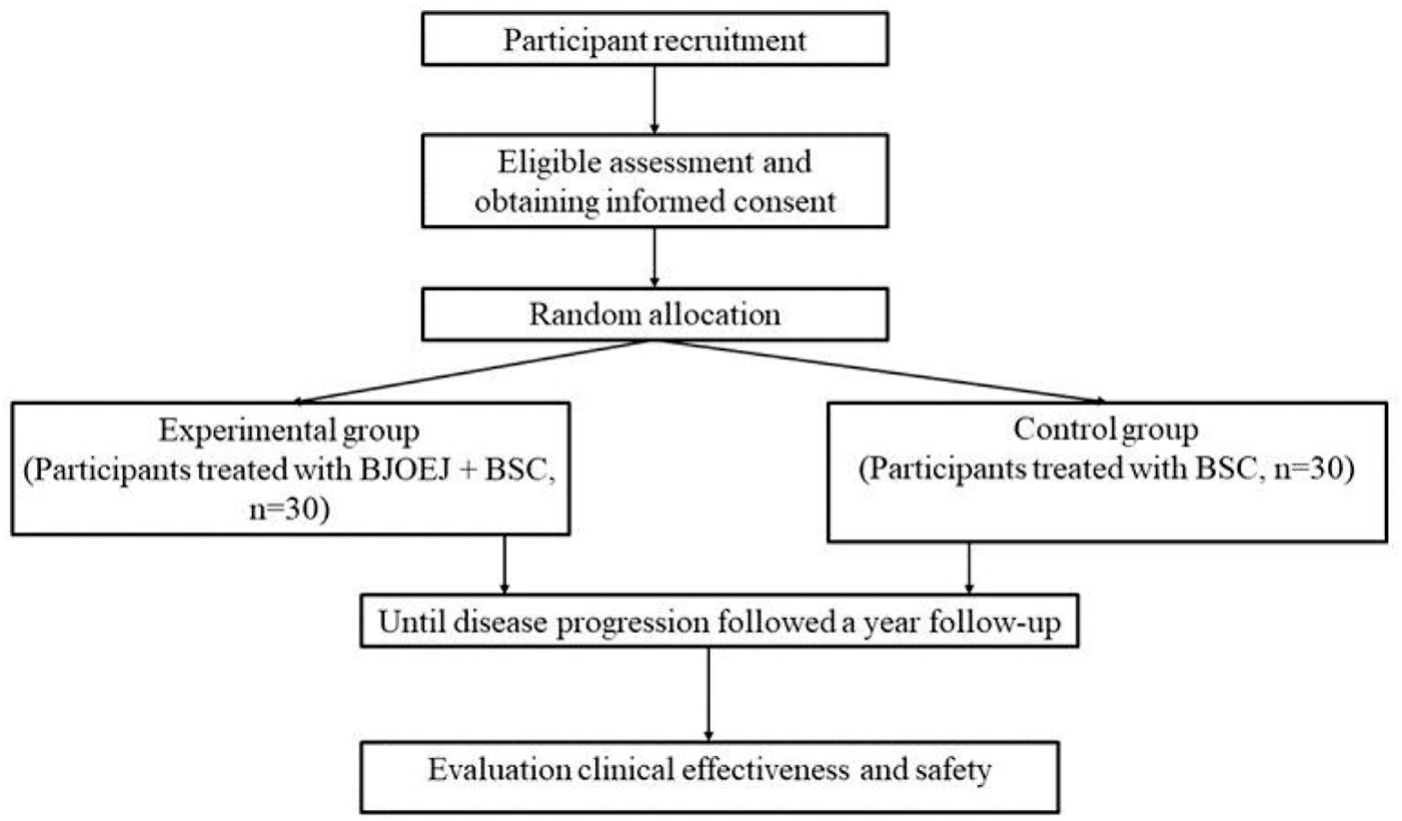
Trial flowchart.

This study is a multicenter, randomized, and controlled clinical study comparing the efficacy and safety of BJOEI combined with BSC versus BSC in patients who are refractory to all available therapy. Eligible patients will be randomized in a 1:1 ratio to either the experimental group or the control group. Both groups will receive BSC in line with the NCCN palliative treatment guidelines (Dans et al., 2021). Participants assigned to the experimental group will be administered 30 mL of intravenous BJOEI daily for 1–14 days in each 28-day cycle. Conversely, the control group will receive only BSC. Treatment for eligible patients will continue until disease progression as defined by the Response Evaluation Criteria in Solid Tumors 1.1 (RECIST 1.1) (Eisenhauer et al., 2009), the onset of unacceptable toxicity, completion of 12 months from randomization, or withdrawal of informed consent. Follow-up evaluations will occur every 2 months until either death, loss of follow-up, or the 12-month mark from randomization. The trial has been registered on the Clinical Trials research platform (ClinicalTrials.gov, ID NCT05897749), and the participant timeline and trial flowchart are displayed in Figures 1, 2, respectively.

### Ethics approval and patient consent

The study protocol has garnered approval from esteemed institutions, including the Guang’anmen Hospital of the China Academy of Chinese Medical Sciences, the First Affiliated Hospital of Guangzhou University of Chinese Medicine, Jiangsu Province Hospital of Chinese Medicine, Yueyang Hospital of Integrated Traditional Chinese and Western Medicine, and the Affiliated Hospital of Shandong University of Traditional Chinese Medicine. Informed consent from the candidates will be obtained by the study investigators at each participating center.

### Outcomes

#### Primary outcome

PFS: PFS is defined as the duration from randomization to the occurrence of tumor progression or death resulting from any cause, whichever transpires first.

#### Secondary outcomes

(1) Objective Response Rate (ORR): ORR represents the percentage of patients achieving a predefined reduction in tumor volume and maintaining this reduction for a specified minimum duration, assessed in accordance with RECIST 1.1 criteria.
(2) OS: OS is the interval from randomization to death caused by any factor.
(3) Quality of Life Assessment: The European Organization for Research and Treatment of Cancer Quality of Life Questionnaire-Core 30 (European Organisation for Research and Treatment of Cancer, 2001) and the Anderson Symptom Assessment Scale (Giesinger et al., 2021) will be employed to assess score changes.

#### Safety outcomes

We will adopt a more rigorous daily monitoring regime throughout the 14-day infusion cycles, incorporating vital signs monitoring, comprehensive clinical symptom assessment, and laboratory test. This enhanced monitoring approach will enable immediate detection and management of infusion-related adverse events, thereby improving patient safety during the most critical treatment periods. Any AEs that arise will be promptly documented, and the physician will provide treatment based on the specific circumstances. Serious AEs will be reported to the ethics committee. Insurance will cover any AEs resulting from the study.

### Eligibility criteria

#### Inclusion criteria

The participants who are included must meet all the following criteria:

(1) Pathologically confirmed stage IV CRC patients who are refractory to all available therapy;
(2) Individuals aged 18 to 75, regardless of gender;
(3) Eastern Cooperative Oncology Group’s (ECOG) performance status is 0–2;
(4) According to RECIST 1.1, at least one measurable target lesion;
(5) Written informed consent obtained.

#### Exclusion criteria

Patients who meet one or more of the following criteria will be excluded:

(1) Brain metastasis, the BRAF-V600E mutation, high microsatellite instability/deficient mismatch repair (MSI-H/dMMR), or NTRK fusion genes;
(2) Patients with a history of other malignancies;
(3) Previously recruited into another drug trial within the last 8 weeks.;
(4) Severe disorders affecting the cardiovascular, cerebrovascular, hepatic, renal, or hematopoietic systems or those whose primary diseases are not effectively controlled;
(5) Pregnant or lactating women, and women not using an effective form of contraception;
(6) History of any hypersensitivity or allergic reaction to BJOEI.
(7) Any condition (e.g. psychological, geographical, etc.) that does not permit compliance with the protocol.

### Randomization, allocation concealment and blinding

#### Randomization strategy

Participants will be randomly assigned to the control or experimental group in a 1:1 ratio using minimization randomization. Given our sample size of 60 patients, we implemented minimization rather than traditional stratified block randomization to ensure balanced allocation across multiple prognostic factors without creating formal strata that might have insufficient sample sizes. The minimization algorithm will consider three key factors: (1) primary tumor location (colon vs. rectal cancer), (2) ECOG performance status (0-1 vs. 2), and (3) study center. This approach is particularly suitable for smaller trials as it ensures balanced allocation across multiple factors while maintaining statistical efficiency.

#### Technical implementation

The randomization process will be overseen by an independent third-party statistical unit, specifically Beijing InnoTech Science & Technology Co., Ltd. A centralized web-based randomization system will implement the minimization algorithm using a computer-generated sequence with a random component (80% deterministic allocation based on minimization, 20% random allocation) to maintain unpredictability while achieving optimal balance. This ratio was selected to optimize both treatment balance and allocation concealment according to established recommendations for minimization algorithms in clinical trials.

#### Allocation concealment

Complete allocation concealment will be maintained through the centralized web-based system, which will only reveal treatment allocation after confirming patient eligibility and completing all required baseline assessments. The randomization sequence will be generated and stored securely by the independent statistical unit, ensuring that investigators and research personnel remain blinded to upcoming assignments throughout the enrollment process.

#### Blinding

This is an open-label study where neither participants nor treating physicians are blinded to treatment assignments due to the nature of the interventions. However, to minimize detection and measurement bias, several measures have been implemented for objective assessment of endpoints. Following CONSORT and SPIRIT guidelines, imaging studies for progression-free survival (PFS) will be centrally reviewed by two blinded board-certified radiologists who remain unaware of treatment assignments. Any discordant evaluations will be resolved by a third independent radiologist who is also blinded to treatment allocation. Quality-of-life assessments will be conducted and analyzed by study coordinators and an external data management team, both unaware of treatment assignments. These measures ensure that both objective and subjective endpoints are assessed impartially, minimizing detection and measurement bias despite the open-label nature of the study.

### Intervention

#### Control group: BSC

BSC is defined as those measures designed to provide palliation of symptoms and improve quality of life as much as possible. All patients will receive BSC based on the NCCN Palliative Treatment Guidelines (Dans et al., 2021).

#### Experimental group: BJOEI, in addition to the BSC

Participants in the experimental group will receive intravenous BJOEI 30 mL per day continuously for 1–14 days of a 28-day cycle.

Both groups will undergo corresponding interventions until disease progression, death, the occurrence of unacceptable toxic effects, withdrawal of consent by the patient, or a decision by the treating physician to discontinue the intervention in the patient’s best interest.

BJOEI (specifications:10mL/tube) will be manufactured by Jiangsu Jiuxu Pharmaceutical Co. Ltd. (Xuzhou, China).

#### Sample size

Many patients experience rapid disease progression with third- and fourth-line therapies, leaving them with limited therapeutic options. BJOEI has shown potential benefits for these patients.As an external pilot RCT, this study is not powered to test efficacy. A total of 60 participants (30 per arm) was selected to provide adequate precision for estimates of median PFS and variance while remaining logistically feasible. This aligns with published recommendations (15–25 per arm) and will inform the definitive phase III sample-size calculation (Sim and Lewis, 2012; Whitehead et al., 2016).

### Data collection and management

The research team will undertake the tasks of data collection and management. Data collection will involve the use of both electronic medical records and case report forms (CRFs). Electronic health records will extract participant demographic details and relevant medical history. Additionally, researchers will systematically accumulate precise individualized data through a meticulously crafted CRF.

Following data collection, designated staff will thoroughly review the CRFs to verify the information. Once finalized, the CRFs will be secured by the head of the research site for safekeeping. After the follow-up period, the original data will be digitized, and the initial CRFs will be sealed and preserved until the designated time for unsealing.

### The trial quality control

The following rules apply to trial quality control.

(1) Each center is required to designate an officer responsible for seamless coordination throughout the entire process.
(2) Participant Protection and Informed Consent;
(3) Strict adherence to the research protocol is imperative throughout the trial;
(4) Ensure Clinical Data Integrity;
(5) Timely Report of Adverse Events.

### Statistical analysis

#### Software and general principles

Data statistical analysis will be conducted using SPSS or SAS software. All analyses will adhere to the intention-to-treat (ITT) principle, encompassing all randomly assigned patients. For participants who withdraw from the trial, their data will be handled using the last observation carried forward (LOCF) method when appropriate.

#### Analysis populations

We have clearly pre-defined the analysis populations as follows:

(1) The modified intention-to-treat (mITT) population will include all randomized patients who receive at least one dose of study treatment (Brucea javanica Oil Emulsion Injection or best supportive care).
(2) The per-protocol (PP) population will comprise patients in the mITT population who complete the study without major protocol deviations, specifically defined as patients who receive ≥80% of planned treatment doses, have no prohibited concomitant medications, and complete all required safety and efficacy assessments per protocol schedule.
(3) The safety set will include all patients who receive at least one dose of study treatment and have at least one post-baseline safety assessment.

#### Statistical description and analysis methods

For continuous variables, normality will be assessed using the Shapiro-Wilk test or Kolmogorov-Smirnov test. If normally distributed, data will be presented as mean ± standard deviation, and between-group comparisons will be performed using independent sample t-tests. If not normally distributed, data will be presented as median and interquartile range (IQR), and the Mann-Whitney U test will be applied for between-group comparisons.

For categorical variables, descriptive statistics will be presented as frequencies and percentages. Between-group comparisons will be performed using the chi-squared test or Fisher’s exact test when the expected frequency in any cell is less than 5.

#### Time-to-event analysis

Kaplan-Meier curves will be used to estimate survival functions for overall survival (OS) and progression-free survival (PFS). The log-rank test will be employed to compare survival curves between treatment groups. Cox proportional-hazards models will be applied to calculate hazard ratios (HRs) with corresponding 95% confidence intervals (CIs), adjusting for pre-specified prognostic factors including ECOG performance status, number of prior treatment lines, and presence of liver metastases. Patients will be censored at the date of last contact for survival analyses and at the date of last tumor assessment for progression-free survival analyses.

#### Primary and secondary analyses

The primary efficacy analysis will be conducted on the mITT population, with supportive analyses performed on the PP population. Safety evaluations will utilize the safety set. The primary endpoint analysis will focus on PFS, while secondary endpoints will include response rate, OS, quality of life measures, and safety parameters.

### Statistical significance

All statistical tests will be two-sided, with a significance level of α = 0.05.

## Discussion

CRC stand as a significant global health burden, ranking as the third most prevalent malignancy. Substantial evidence indicates that 90% of CRC cases are diagnosed in individuals aged 50 and above. Alarmingly, the incidence of CRC among younger populations is increasing (Penz et al., 2023). The 5-year survival rate for mCRC remains dishearteningly low at 6% (Coco et al., 2012). The most effective treatment for mCRC is 5-FU-based regimens. However, disease progression after two lines of doublet chemotherapy remains inevitable. Although several drugs are the standard third-line treatment of mCRC in guidelines (Grothey et al., 2013; Dasari et al., 2023; Mayer et al., 2015), they yield minimal response rate and short PFS and OS. In general, the efficacy of third-line and subsequent treatments is unsatisfactory. Few treatment options exist for this population of patients. Traditional Chinese Medicine (TCM) theories and treatment approaches are gaining global acceptance, with integrative Chinese and Western medicine emerging as a novel avenue in cancer therapy. TCM demonstrates unique advantages in tumor treatment. BJOEI have been widely used for the treatment of cancer in China. Numerous clinical studies have shown that BJOEI when combined with FOLFOX/FOLFIRI regimens, enhances tumor treatment efficacy, improves patients' quality of life, and mitigates adverse reactions (Gao and Zhang, 2023).

In prior studies of BJOEI on CRC, the primary endpoint was the ORR. However, this measure alone cannot fully capture the clinical benefits of the trial drug. PFS is utilized both as a surrogate end-point for OS and as a primary trial end-point in itself (Robinson et al., 2014). Therefore, we hope to further objectively evaluate the clinical effectiveness of BJOEI combined with BSC from the PFS status. In addition, Safety assessments will include the incidence, nature, and severity of adverse events; serious adverse events and laboratory abnormalities. Laboratory safety assessments will include the regular monitoring of hematology and blood chemistry.

However, the study design has several limitations. Firstly, obtaining a placebo for BJOEI in China is challenging, making it impossible to blind the study. Secondly, this study is a small-scale trial intended to provide effect size data for calculating sample sizes in future large-scale randomized controlled trials. Thirdly, since the experiment will be conducted in five centers within China, we now clearly state that the findings may primarily reflect outcomes in the Chinese population and that caution should be exercised when extrapolating results to other ethnic groups due to potential pharmacogenomic differences.

The present findings will be hypothesis-generating and will supply the parameters necessary for a rigorously powered confirmatory phase III trial. We anticipate that this trial will offer a new treatment option for advanced CRC patients who are unresponsive to standard therapies.
