# Supplementary material for: Brucea javanica oil emulsion plus supportive care for refractory advanced colorectal cancer: a pilot RCT protocol
Source: Front Pharmacol. 2025 Jul 21;16:1610575. doi: 10.3389/fphar.2025.1610575 (PMC12318930; doi:10.3389/fphar.2025.1610575)
Supplement: Supplementary file 1 [file DataSheet1.pdf]

Reporting checklist for protocol of a clinical trial.

Based on the SPIRIT guidelines.

Instructions to authors

Complete this checklist by entering the page numbers from your manuscript where readers will find each of the items listed below.

Your article may not currently address all the items on the checklist. Please modify your text to include the missing information. If you are certain that an item does not apply, please write "n/a" and provide a short explanation.

Upload your completed checklist as an extra file when you submit to a journal.

In your methods section, say that you used the SPIRITreporting guidelines, and cite them as:

Chan A-W, Tetzlaff JM, Gøtzsche PC, Altman DG, Mann H, Berlin J, Dickersin K, Hróbjartsson A, Schulz KF, Parulekar WR, Krleža-Jerić K, Laupacis A, Moher D. SPIRIT 2013 Explanation and Elaboration: Guidance for protocols of clinical trials. BMJ. 2013;346:e7586

		Reporting Item	Page Number
**Administrative information**			
Title	#1	Descriptive title identifying the study design, population, interventions, and, if applicable, trial acronym	Title
Trial registration	#2a	Trial identifier and registry name. If not yet registered, name of intended registry	Trial registration
Trial registration: data set	#2b	All items from the World Health Organization Trial Registration Data Set	n/a
Protocol version	#3	Date and version identifier	n/a
Funding	#4	Sources and types of financial, material, and other support	Funding
Roles and responsibilities: contributorship	#5a	Names, affiliations, and roles of protocol contributors	Contributors

Roles and responsibilities: sponsor contact information	#5b	Name and contact information for the trial sponsor	Contributors
Roles and responsibilities: sponsor and funder	#5c	Role of study sponsor and funders, if any, in study design; collection, management, analysis, and interpretation of data; writing of the report; and the decision to submit the report for publication, including whether they will have ultimate authority over any of these activities	Ethics approval and patient consent
Roles and responsibilities: committees	#5d	Composition, roles, and responsibilities of the coordinating centre, steering committee, endpoint adjudication committee, data management team, and other individuals or groups overseeing the trial, if applicable (see Item 21a for data monitoring committee)	Ethics approval and patient consent
**Introduction**			
Background and rationale	#6a	Description of research question and justification for undertaking the trial, including summary of relevant studies (published and unpublished) examining benefits and harms for each intervention	Introduction
Background and rationale: choice of comparators	#6b	Explanation for choice of comparators	Introduction
Objectives	#7	Specific objectives or hypotheses	Introduction
Trial design	#8	Description of trial design including type of trial (eg, parallel group, crossover, factorial, single group), allocation ratio, and framework (eg, superiority, equivalence, non-inferiority, exploratory)	Introduction
**Methods: Participants, interventions, and outcomes**			

Study setting	#9	Description of study settings (eg, community clinic, academic hospital) and list of countries where data will be collected. Reference to where list of study sites can be obtained	Study design
Eligibility criteria	#10	Inclusion and exclusion criteria for participants. If applicable, eligibility criteria for study centres and individuals who will perform the interventions (eg, surgeons, psychotherapists)	Eligibility criteria
Interventions: description	#11a	Interventions for each group with sufficient detail to allow replication, including how and when they will be administered	Intervention
Interventions: modifications	#11b	Criteria for discontinuing or modifying allocated interventions for a given trial participant (eg, drug dose change in response to harms, participant request, or improving / worsening disease)	Intervention
Interventions: adherance	#11c	Strategies to improve adherence to intervention protocols, and any procedures for monitoring adherence (eg, drug tablet return; laboratory tests)	Intervention
Interventions: concomitant care	#11d	Relevant concomitant care and interventions that are permitted or prohibited during the trial	Intervention
Outcomes	#12	Primary, secondary, and other outcomes, including the specific measurement variable (eg, systolic blood pressure), analysis metric (eg, change from baseline, final value, time to event), method of aggregation (eg, median, proportion), and time point for each outcome. Explanation of the clinical relevance of chosen efficacy and harm outcomes is strongly recommended	Outcomes
Participant timeline	#13	Time schedule of enrolment, interventions (including any run-ins and washouts), assessments, and visits for participants. A schematic diagram is highly recommended (see Figure)	Study design
Sample size	#14	Estimated number of participants needed to	Sample size

		achieve study objectives and how it was determined, including clinical and statistical assumptions supporting any sample size calculations	
Recruitment	#15	Strategies for achieving adequate participant enrolment to reach target sample size	Study design
**Methods: Assignment of interventions (for controlled trials)**			
Allocation: sequence generation	#16a	Method of generating the allocation sequence (eg, computer-generated random numbers), and list of any factors for stratification. To reduce predictability of a random sequence, details of any planned restriction (eg, blocking) should be provided in a separate document that is unavailable to those who enrol participants or assign interventions	Randomization, allocation concealment and blinding
Allocation concealment mechanism	#16b	Mechanism of implementing the allocation sequence (eg, central telephone; sequentially numbered, opaque, sealed envelopes), describing any steps to conceal the sequence until interventions are assigned	Randomization, allocation concealment and blinding
Allocation: implementation	#16c	Who will generate the allocation sequence, who will enrol participants, and who will assign participants to interventions	Randomization, allocation concealment and blinding
Blinding (masking)	#17a	Who will be blinded after assignment to interventions (eg, trial participants, care providers, outcome assessors, data analysts), and how	Randomization, allocation concealment and blinding
Blinding (masking): emergency unblinding	#17b	If blinded, circumstances under which unblinding is permissible, and procedure for revealing a participant’s allocated intervention during the trial	Randomization, allocation concealment and blinding

**Methods: Data collection, management, and analysis**			
Data collection plan	#18a	Plans for assessment and collection of outcome, baseline, and other trial data, including any related processes to promote data quality (eg, duplicate measurements, training of assessors) and a description of study instruments (eg, questionnaires, laboratory tests) along with their reliability and validity, if known. Reference to where data collection forms can be found, if not in the protocol	Data collection and management
Data collection plan: retention	#18b	Plans to promote participant retention and complete follow-up, including list of any outcome data to be collected for participants who discontinue or deviate from intervention protocols	Data collection and management
Data management	#19	Plans for data entry, coding, security, and storage, including any related processes to promote data quality (eg, double data entry; range checks for data values). Reference to where details of data management procedures can be found, if not in the protocol	Data collection and management
Statistics: outcomes	#20a	Statistical methods for analysing primary and secondary outcomes. Reference to where other details of the statistical analysis plan can be found, if not in the protocol	Statistical analysis
Statistics: additional analyses	#20b	Methods for any additional analyses (eg, subgroup and adjusted analyses)	n/a
Statistics: analysis population and missing data	#20c	Definition of analysis population relating to protocol non-adherence (eg, as randomised analysis), and any statistical methods to handle missing data (eg, multiple imputation)	Statistical analysis
**Methods: Monitoring**			

Data monitoring: formal committee	#21a	Composition of data monitoring committee (DMC); summary of its role and reporting structure; statement of whether it is independent from the sponsor and competing interests; and reference to where further details about its charter can be found, if not in the protocol. Alternatively, an explanation of why a DMC is not needed	The trial quality control
Data monitoring: interim analysis	#21b	Description of any interim analyses and stopping guidelines, including who will have access to these interim results and make the final decision to terminate the trial	The trial quality control
Harms	#22	Plans for collecting, assessing, reporting, and managing solicited and spontaneously reported adverse events and other unintended effects of trial interventions or trial conduct	The trial quality control
Auditing	#23	Frequency and procedures for auditing trial conduct, if any, and whether the process will be independent from investigators and the sponsor	The trial quality control
**Ethics and dissemination**			
Research ethics approval	#24	Plans for seeking research ethics committee / institutional review board (REC / IRB) approval	The trial quality control
Protocol amendments	#25	Plans for communicating important protocol modifications (eg, changes to eligibility criteria, outcomes, analyses) to relevant parties (eg, investigators, REC / IRBs, trial participants, trial registries, journals, regulators)	The trial quality control
Consent or assent	#26a	Who will obtain informed consent or assent from potential trial participants or authorised surrogates, and how (see Item 32)	Ethics approval and patient consent
Consent or assent: ancillary studies	#26b	Additional consent provisions for collection and use of participant data and biological specimens in ancillary studies, if applicable	n/a

Confidentiality	#27	How personal information about potential and enrolled participants will be collected, shared, and maintained in order to protect confidentiality before, during, and after the trial	Ethics approval and patient consent
Declaration of interests	#28	Financial and other competing interests for principal investigators for the overall trial and each study site	Competing interests
Data access	#29	Statement of who will have access to the final trial dataset, and disclosure of contractual agreements that limit such access for investigators	Data availability statement
Ancillary and post trial care	#30	Provisions, if any, for ancillary and post-trial care, and for compensation to those who suffer harm from trial participation	n/a
Dissemination policy: trial results	#31a	Plans for investigators and sponsor to communicate trial results to participants, healthcare professionals, the public, and other relevant groups (eg, via publication, reporting in results databases, or other data sharing arrangements), including any publication restrictions	Data availability statement
Dissemination policy: authorship	#31b	Authorship eligibility guidelines and any intended use of professional writers	Data availability statement
Dissemination policy: reproducible research	#31c	Plans, if any, for granting public access to the full protocol, participant-level dataset, and statistical code	Data availability statement
**Appendices**			
Informed consent materials	#32	Model consent form and other related documentation given to participants and authorised surrogates	Ethics approval and patient consent
Biological specimens	#33	Plans for collection, laboratory evaluation, and storage of biological specimens for genetic or molecular analysis in the current trial and for future use in ancillary studies, if applicable	n/a

None The SPIRIT Explanation and Elaboration paper is distributed under the terms of the Creative Commons Attribution License CC-BY-NC. This checklist can be completed online using https://www.goodreports.org/, a tool made by the EQUATOR Network in collaboration with Penelope.ai

伦理审查批件

项目名称	鸦胆子油乳注射液对多线治疗失败的晚期结直肠癌患者生存期干预的临床研究		
批件号	2022-269-KY	项目来源	省部级课题
研究单位	中国中医科学院广安门医院		
申办者	无		
主要研究者	张英	研究科室	肿瘤科
审查类别	初始审查	审查方式	会议审查
审查日期	2022/12/21	审查地点	中国中医科学院广安门医院
审查委员	王笑频，杨睿，梁军，郭敬，杨响光，宋庆桥，沈瑞英，刘新敏，崔全起，隋昕，白煜，边永君，韩梅，王保华		
批准文件及版本	1. 研究方案（版本号:v1.0 版本日期:2022-11-15）<br>2. 病例报告表（版本号:1.0 版本日期:2022-11-15）<br>3. 知情同意书（版本号:v1.0 版本日期:2022-11-15）		
审查意见	根据国家卫生计生委《涉及人的生物医学研究伦理审查办法》、国家药品食品监督管理局《药物临床试验质量管理规范》、《医疗器械临床试验规定》、《药物临床试验伦理审查工作指导原则》国家中医药管理局《中医药临床研究伦理审查管理规范》以及《赫尔辛基宣言》和国际医学科学组织委员会颁布的《人体生物医学研究国际道德指南》的伦理原则，经本伦理委员会审查，同意按所批准的临床研究方案、知情同意书等材料开展本项研究。<br>请遵循GCP原则、遵循伦理委员会批准的方案开展临床研究，保护受试者的健康与权利。<br>若在3年内未启动研究，本批件作废，需重新提交伦理审查申请。<br>研究开始前，请申请人完成临床试验注册。涉及中国人类遗传资源、需要报批的研究项目，需在获得中国人类遗传资源管理办公室批准后才能开始研究。<br>研究过程中若变更主要研究者，对临床研究方案、知情同意书、招募材料等的任何修改，请申请人提交修正案审查申请。<br>如发生严重不良事件以及影响研究风险受益比的非预期不良事件，请申请人在获知后15天内提交严重不良事件报告，如果是致死或危及生命的非预期严重不良反应，请在首次获知后7天内提交严重不良事件报告，并在随后的8天内报告、完善随访信息。<br>请按照伦理委员会规定的年度/定期跟踪审查频率，申请人在截止日期前1个月提交研究进展报告；申办者应当向组长单位伦理委员会提交各中心研究进展的汇总报告；当出现任何可能显著影响试验进行或增加受试者危险的情况时，请申请人及时向伦理委员会提交书面报告。<br>研究纳入了不符合纳入标准或符合排除标准的受试者，符合中止试验规定而		

	未让受试者退出研究，给予错误治疗或剂量，给予方案禁止的合并用药等没有遵从方案开展研究的情况；或可能对受试者的权益/健康以及研究的科学性造成不良影响等违背GCP原则的情况，请申办者/监查员/研究者提交违背方案报告。<br>提前终止或完成临床研究，请及时提交研究完成报告。		
批件有效期	2022/12/26 ~2025/12/31		
跟踪审查频率	12个月	跟踪审查截止日期	2023/12/25
联系人与联系方式	联系人：乔洁 联系电话：010-88001552 Email：gamhec@126.com		
主任委员/副主任委员签字			

中国中医科学院广安门医院伦理委员会（盖章）

日期: 2022/12/26

鸦胆子油乳对多线治疗失败的晚期结直肠癌患者生存期干预的临床研究

研究方案摘要

**项目名称**	鸦胆子油乳注射液对多线治疗失败的晚期结直肠癌患者生存期干预的临床研究。
**研究目的**	初步收集鸦胆子油乳注射液联合最佳支持治疗对多线治疗失败的晚期结直肠癌的相关数据，为较大规模临床实验提供数据支持。
**研究设计**	前瞻性、多中心、随机对照临床研究
**病例选择**	**纳入标准**<br>① 经病理学确诊的 IV 期三线及三线以上治疗失败的结直肠癌患者；<br>② 年龄 18-75 岁，男女不限；<br>③ ECOG 评分 0-2 分；<br>④ 按 RECIST1.1 标准，至少具有一个可测量病灶；<br>⑤ 自愿加入研究、签署知情同意书，依从性好配合随访。
	**排除标准**<br>① 伴有脑转移、BRAF-V600E 突变、MSI-H/dMMR、NTRK 融合基因的患者；<br>② 合并其他部位原发肿瘤者；<br>③ 妊娠或哺乳期妇女；育龄期女性及其配偶在临床研究期间及结束后 6 个月内不能采取有效的避孕措施；<br>④合并严重的心、脑血管、肝肾、造血系统功能障碍或原发性疾病未有效控制者；<br>⑤ 过去 8 周内参加过其它药物临床实验；<br>⑥ 已知对研究药物过敏或者不能耐受的患者。

<table>
<tr><td>治疗
方案</td><td>试验组：鸦胆子油乳注射液联合最佳支持治疗
对照组：最佳支持治疗
给药方法：
鸦胆子油乳注射液：静脉滴注，每次 30ml（加灭菌生理盐水 250ml），每日 1 次，在 28 天内连续应用 14 天为一个治疗周期。
最佳支持治疗：根据 NCCN 最佳姑息性治疗指南（2022.V1），给予最佳支持治疗。最佳支持治疗包括：
（1）疼痛管理：准确完善疼痛评估，综合合理措施治疗疼痛，按照疼痛三阶梯治疗原则进行，积极预防处理止痛药物的不良反应，同时关注病因治疗。重视病人及家属疼痛教育和社会精神心理支持，加强沟通随访；
（2）营养支持：常规评估营养状态，给予适当的营养支持，倡导肠内营养支持；
（3）精神心理干预：癌症心理专业医师进行心理干预和必要的精神药物干预；
（4）转移部位干预；对于肺、肝、腹膜等部位的转移灶需要在多学科讨论的模式下进行综合治疗。</td></tr>
<tr><td>疗效
评定</td><td>主要目标：无进展生存期。
次要目标：客观有效率、总生存期、生活质量评分。</td></tr>
</table>

统计<br>方法	**统计分析计划**<br>数据的统计分析将使用 SPSS 或 SAS 软件进行。所有分析将遵循意向性治疗原则，涵盖所有随机分配的患者。对于退出试验的参与者，其数据将采用最后观察值前移法处理。主要分析将集中在意向性患者中，而安全性评估将使用一个安全集。<br>计量资料符合正态分布的采用均数±标准差进行统计描述，不符合正态分布的采用中位数和四分位数间距进行统计描述。两组间比较符合正态分布者使用两独立样本 t 检验，不符合正态分布者应用秩和检验（Mann Whitney U 检验）；计数资料采用频数进行统计描述。两组间比较采用卡方检验或 Fisher 确切概率法；对于无进展生存期和总生存期的生存分析采用 Kaplan-Meier 方法，这是一种非参数统计方法，用于描述生存时间的分布。
研究<br>期限	2022 年 12 月－2025 年 12 月

Clinical Investigation of Yadanzi Oil Emulsion as a Survival Intervention in Patients with Advanced Colorectal Cancer Resistant to Multiple Lines of Therapy

1.Study Objectives

The primary objective of this study is to gather preliminary data to inform a phase III clinical trial evaluating the efficacy and safety of BJOEI in combination with best supportive care for patients with advanced colorectal cancer who are refractory to all existing therapies.

2.Study design

This study is a multicenter, randomized, and controlled clinical study.

3.Eligibility criteria

3.1Inclusion criteria

The participants who are included must meet all the following criteria:

(1) Pathologically confirmed stage IV CRC patients who are refractory to all available therapy;

(2) Individuals aged 18 to 75, regardless of gender;

(3) Eastern Cooperative Oncology Group's (ECOG) performance status is 0 to 2;

(4) According to RECIST 1.1, at least one measurable target lesion;

(5) Written informed consent obtained.

3.2Exclusion criteria

Patients who meet one or more of the following criteria will be excluded:

(1) Brain metastasis, the BRAF-V600E mutation, high microsatellite instability/deficient mismatch repair (MSI-H/dMMR), or NTRK fusion genes;

(2) Patients with a history of other malignancies;

(3) Previously recruited into another drug trial within the last 8 weeks.;

(4) Severe disorders affecting the cardiovascular, cerebrovascular, hepatic, renal, or hematopoietic systems or those whose primary diseases are not effectively controlled;

(5) Pregnant or lactating women, and women not using an effective form of

contraception;

(6) History of any hypersensitivity or allergic reaction to BJOEI.

4.Intervention

4.1Control Group: best supportive care (BSC)

BSC is defined as those measures designed to provide palliation of symptoms and improve quality of life as much as possible. All patients will receive BSC based on the NCCN Palliative Treatment Guidelines.

4.2Experimental Group: BJOEI, in addition to the BSC

Participants in the experimental group will receive intravenous BJOEI 30 ml per day continuously for 1-14 days of a 28-day cycle.

4.3Best supportive care encompasses:

(1) Pain Management: A comprehensive and precise assessment of pain is paramount, followed by integrated and judicious therapeutic strategies adhering to the three-step analgesic ladder. Proactive measures should be taken to prevent and address adverse reactions to analgesics, alongside etiological treatment. Emphasizing patient and family education regarding pain management, along with societal, spiritual, and psychological support, is crucial. Enhanced communication and continuous follow-up are essential.

(2) Nutritional Support: Routine assessment of nutritional status should be conducted to provide appropriate nutritional interventions, with a strong advocacy for enteral nutrition.

(3) Psychological Interventions: Psychological support should be administered by specialized oncology psychologists, including necessary psychiatric pharmacological interventions.

(4) Metastatic Site Interventions: For metastases in the lungs, liver, or peritoneum, a multidisciplinary approach should be employed to devise comprehensive treatment strategies.

5.Outcomes

5.1Primary outcome:

PFS: PFS is defined as the duration from randomization to the occurrence of tumor

progression or death resulting from any cause, whichever transpires first.

5.2Secondary outcomes:

(1) Objective Response Rate (ORR): ORR represents the percentage of patients achieving a predefined reduction in tumor volume and maintaining this reduction for a specified minimum duration, assessed in accordance with RECIST 1.1 criteria.

(2) OS: OS is the interval from randomization to death caused by any factor.

(3) Quality of Life Assessment: The European Organization for Research and Treatment of Cancer Quality of Life Questionnaire-Core 30 and the Anderson Symptom Assessment Scale will be employed to assess score changes.

6.Statistical analysis

Data statistical analysis will be conducted using SPSS or SAS software. All analyses will adhere to the intention-to-treat (ITT) principle, encompassing all randomly assigned patients. For participants who withdraw from the trial, their data will be handled using the last observation carried forward method. The primary analysis will focus on the ITT population, while safety evaluations will utilize a safety set.

For measurement data, if normally distributed, mean ± standard deviation will be used for statistical description; if not normally distributed, median and interquartile range will be employed. Comparative analysis between groups for normally distributed data will use independent sample t-tests; for non-normally distributed data, the Mann-Whitney U test will be applied. Frequency will be used for descriptive statistics of categorical data. Comparisons between groups will be performed using the chi-squared test or Fisher's exact test. Survival analysis for progression-free survival and overall survival will utilize the Kaplan-Meier method, a non-parametric technique for delineating the distribution of survival times.

7.Study period

The research period is from December 2022 to December 2025
